# Estimated Incidence of Antimicrobial Drug–Resistant Nontyphoidal *Salmonella* Infections, United States, 2004–2012

**DOI:** 10.3201/eid2301.160771

**Published:** 2017-01

**Authors:** Felicita Medalla, Weidong Gu, Barbara E. Mahon, Michael Judd, Jason Folster, Patricia M. Griffin, Robert M. Hoekstra

**Affiliations:** Centers for Disease Control and Prevention, Atlanta, Georgia, USA

**Keywords:** *Salmonella*, antimicrobial resistance, antibiotic resistance, drug resistance, ceftriaxone, ciprofloxacin, ampicillin, incidence, bacteria, food safety, United States

## Abstract

*Salmonella* infections are a major cause of illness in the United States. The antimicrobial agents used to treat severe infections include ceftriaxone, ciprofloxacin, and ampicillin. Antimicrobial drug resistance has been associated with adverse clinical outcomes. To estimate the incidence of resistant culture-confirmed nontyphoidal *Salmonella* infections, we used Bayesian hierarchical models of 2004–2012 data from the Centers for Disease Control and Prevention National Antimicrobial Resistance Monitoring System and Laboratory-based Enteric Disease Surveillance. We based 3 mutually exclusive resistance categories on susceptibility testing: ceftriaxone and ampicillin resistant, ciprofloxacin nonsusceptible but ceftriaxone susceptible, and ampicillin resistant but ceftriaxone and ciprofloxacin susceptible. We estimated the overall incidence of resistant infections as 1.07/100,000 person-years for ampicillin-only resistance, 0.51/100,000 person-years for ceftriaxone and ampicillin resistance, and 0.35/100,000 person-years for ciprofloxacin nonsusceptibility, or ≈6,200 resistant culture-confirmed infections annually. These national estimates help define the magnitude of the resistance problem so that control measures can be appropriately targeted.

Each year in the United States, nontyphoidal *Salmonella* causes an estimated 1.2 million illnesses, 23,000 hospitalizations, and 450 deaths (*1*). Antimicrobial drug–resistant *Salmonella* is a serious threat to public health (*2*). *Salmonella* infections have been linked to a variety of sources, particularly foods of animal origin (e.g., beef, poultry, eggs, dairy products) and produce (*3*–*5*). Most antimicrobial drug–resistant nontyphoidal *Salmonella* infections are caused by 4 of the 5 serotypes most commonly isolated during 2004–2012: Typhimurium, Enteritidis, Newport, and Heidelberg (*6**–**10*). The predominance of these 4 serotypes reflects their ability to persist in food animals, be transmitted through the food supply, and cause illness in humans (*10*,*11*).

Most nontyphoidal *Salmonella* infections do not require antimicrobial treatment. However, treatment is recommended for severe infections, including invasive illnesses such as bacteremia and meningitis (*12*). Third-generation cephalosporins (e.g., ceftriaxone) and fluoroquinolones (e.g., ciprofloxacin) are empirically used to treat severe nontyphoidal *Salmonella* infections. Because fluoroquinolones are not routinely prescribed for children, third-generation cephalosporins are particularly important for use in children. Ampicillin remains a useful agent for treating infections documented as susceptible (*12*–*14*). Adverse clinical outcomes (e.g., increased rates of hospitalization, bloodstream infection, invasive illness, and death) have been associated with resistant infections, and treatment failures have been reported for infections with reduced susceptibility to ciprofloxacin (*5*,*15*–*19*).

Estimates of the incidence of resistant *Salmonella* infections are needed to inform policy decisions. The National Antimicrobial Resistance Monitoring System (NARMS) monitors resistance among salmonellae by testing samples of isolates from ill persons and determining the percentage of isolates that display resistance (*8*,*9*). For extrapolation from resistance percentages to incidence of resistant infections, the incidence of *Salmonella* infections must be known. *Salmonella* incidence data for this calculation are provided by the National Laboratory-based Enteric Disease Surveillance (LEDS) system (*6*). Serotype Heidelberg provides an illustration of why estimates of the incidence of resistant infections are needed. During 2004–2012, the percentage of ceftriaxone-resistant isolates increased from 9% to 22% (*8*,*9*). At the same time, the incidence of Heidelberg infections declined from 0.60 to 0.31 infections/100,000 population (*6*). Thus, to assess whether the incidence of resistant Heidelberg infections is changing, estimates of the incidence of resistant Heidelberg infections are needed. Using Bayesian hierarchical models of resistance percentages and *Salmonella* incidence with data from the 2 surveillance systems, we estimated the incidence of culture-confirmed infections caused by nontyphoidal *Salmonella* with resistance to ceftriaxone, nonsusceptibility to ciprofloxacin, and resistance to ampicillin and provide such estimates for major serotypes (*20*). We describe this modeling approach of combining data from the 2 systems to obtain improved estimates and measures of uncertainties.

## Methods

### LEDS 

Clinical laboratories send *Salmonella* isolated from humans to public health laboratories in 50 states and many local health departments for serotyping (*6*). Culture-confirmed *Salmonella* isolates are reported to the Centers for Disease Control and Prevention (CDC) through LEDS (*6*). Excluded from this report are serotypes Typhi and Paratyphi, for which the only reservoir is humans and which account for <1% of *Salmonella* infections in the United States (*6*,*11*,*12*). Hereafter, we use the term *Salmonella* to refer to nontyphoidal *Salmonella*.

### NARMS 

NARMS is a collaboration among CDC, the US Food and Drug Administration (FDA), the US Department of Agriculture, and state and local health departments. NARMS monitors resistance among enteric bacteria isolated from humans, retail meat, and food animals (*8*,*9*). Public health laboratories of 50 state and 4 local health departments submit a subset (every 20th) of *Salmonella* isolates that they receive from clinical laboratories to the CDC NARMS for susceptibility testing (*8*,*9*).

From 2004 through 2012, CDC tested *Salmonella* isolates for susceptibility to agents representing 8–9 classes of antimicrobial agents. MICs were determined by broth microdilution (Sensititer; Trek Diagnostics, Westlake, OH, USA) and interpreted by using criteria from the Clinical and Laboratory Standards Institute when available (*8*,*19*). We defined ceftriaxone resistance as MIC >4 μg/mL, ampicillin resistance as MIC >32 μg/mL, and nonsusceptibility to ciprofloxacin as MIC >0.12 μg/mL; the latter includes resistant and intermediate categories defined by the Clinical and Laboratory Standards Institute (*8*,*19*).

### Resistance Categories for Estimation of Resistance Incidence 

**Figure 1:**
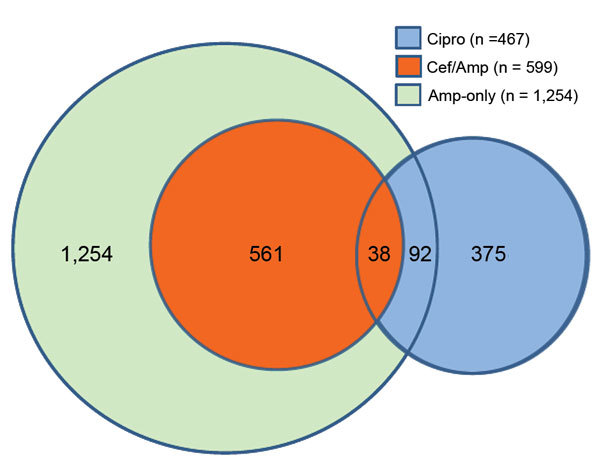
Number of nontyphoidal *Salmonella* isolates with clinically important resistance, by resistance category, United States, 2004–2012. Three mutually exclusive categories were defined. Isolates in each category may have resistance to other agents: 99% of the 599 Cef/Amp isolates, 43% of the 467 Cipro isolates, and 89% of the 1,254 Amp-only isolates were resistant to >1 antimicrobial class other than cephems, quinolones, or penicillins. Amp-only, resistant to ampicillin but susceptible to ceftriaxone and ciprofloxacin; Cef/Amp, resistant to ceftriaxone (MIC >4 μg/mL) and ampicillin (MIC >32 μg/mL); Cipro, nonsusceptible to ciprofloxacin (MIC >0.12 μg /mL) but susceptible to ceftriaxone; NTS, nontyphoidal *Salmonella.*

We defined 3 mutually exclusive categories of clinically important resistance according to results of testing for ceftriaxone, ciprofloxacin, and ampicillin (Figure 1) (*8*,*19*): ceftriaxone/ampicillin resistance indicates resistance to ceftriaxone and ampicillin (because all ceftriaxone-resistant isolates are ampicillin resistant); ciprofloxacin nonsusceptibility indicates nonsusceptibility to ciprofloxacin but susceptibility to ceftriaxone; and ampicillin-only resistance indicates resistance to ampicillin but susceptibility to ceftriaxone and ciprofloxacin. Isolates in each category may be resistant to other agents. Hereafter, we refer to any resistance included in any of these 3 clinically important categories as overall resistance. Unlike the 2013 CDC report, which includes estimates for resistance to >5 antimicrobial drug classes, we focused on the 3 agents used to treat invasive infections (*2*).

### Bayesian Hierarchical Model 

We used 2004–2012 data from NARMS, LEDS, and the US Census Bureau as input in the Bayesian hierarchical model (*6*,*8*,*21*). From NARMS, we used resistance proportions calculated as the number of resistant isolates divided by the number of isolates tested per state and year (state-year). We included only fully serotyped isolates. From LEDS, we used the number of culture-confirmed infections reported for state-year. We included all LEDS isolates; for each state, the serotypes of nonserotyped and partially serotyped isolates were imputed on the basis of the observed proportions of 5 serotype categories (Typhimurium, Enteritidis, Newport, Heidelberg, and other) among fully serotyped isolates over the 9 years. We used US Census population data for each state-year to express incidence (infections per 100,000 persons per year [person-years]).

In the Bayesian hierarchical model, we assumed normal distribution for LEDS *Salmonella* incidence data and binomial distribution for NARMS data. The Bayesian hierarchical model of *Salmonella* incidence and resistance data incorporated state, year, and state-year interaction effects. State and year effects used borrowed strength from contiguous states and previous years. Borrowed strength refers to the idea that quantities of interest are related to each other, and information on one can provide information on another (*22*). We excluded Alaska and Hawaii because they are distant from the 48 contiguous states and so the Bayesian hierarchical model could not be well applied. We excluded the District of Columbia because it did not begin submitting isolates to NARMS until 2008 (*9*). In preliminary analyses, we reviewed LEDS *Salmonella* incidence data by state-year to identify outliers that may need modeling adjustments, knowing that some states do not routinely receive all isolates from clinical laboratories (*6*). The models are described in the Technical Appendix.

We generated Bayesian hierarchical model posterior estimates of *Salmonella* infection incidence rates, resistance proportions, and resistant infection incidence rates (resistance incidence) by state-year for each of the 5 serotype categories by using Markov chain Monte Carlo simulations (*22*–*24*). State-year resistance incidence estimates, expressed per 100,000 person-years, were calculated as follows: ([estimated number of infections for state-year/census population for state-year] × 100,000) × (estimated resistance proportion for state-year). We calculated the means of the 48 state-year mean posterior estimates for each of the 9 study years. We generated overall estimates for 2004–2012 by calculating means and 95% credible intervals (CrIs) from the 9-year mean estimates. We used 2.5th and 97.5th percentiles of 5,000 samples of posterior estimates for the 95% CrIs. For each of the 5 serotype categories, we estimated resistance incidence for the mutually exclusive categories and derived overall resistance incidence estimates by summing them. For all *Salmonella*, estimates were calculated by summing estimates derived for the 5 serotype categories.

As part of model fitting, we plotted observed versus Bayesian hierarchical model–derived (predicted) estimates of *Salmonella* infection incidence, resistance proportion, and resistance incidence by state-year for the 5 serotype categories by resistance category. We assessed the shrinkage of resistance proportions (observed vs. predicted values) related to the number of isolates tested; shrinkage refers to an estimation scheme that borrows strength from related quantities to adjust individual estimates (online Technical Appendix) (*25*). To assess fluctuations over the 9 years of the study, we derived mean estimates and 95% CrIs for 3-year periods (2004–2006, 2007–2009, and 2010–2012) by using an even split of time for simplicity.

## Results

### Overall *Salmonella* Infection and Resistance Surveillance Data

From 2004 through 2012, the 48 contiguous states reported 369,254 culture-confirmed *Salmonella* infections to LEDS. The periods 2004–2006, 2007–2009, and 2010–2012 accounted for 30%, 33%, and 37% of infections, respectively. Among the isolates from these infections, 87% were serotyped as follows: Enteritidis (19%), Typhimurium (18%), Newport (11%), Heidelberg (4%), and all other serotypes (48%). The remaining 13% were not fully serotyped. These 4 primary serotypes, which were among the 5 most commonly reported to LEDS overall, accounted for 52% of fully serotyped isolates. Of the 48 states, <2% of isolates were not fully serotyped for 10 states, 2%–10% for 27 states, 11%–29% for 5 states, and >62% for 6 states.

From 2004 through 2012, NARMS tested 19,410 *Salmonella* isolates from the 48 states for resistance. The periods 2004–2006, 2007–2009, and 2010–2012 accounted for 30%, 34%, and 36% of isolates, respectively. Most (98%) were fully serotyped as follows: Enteritidis (18%), Typhimurium (17%), Newport (11%), Heidelberg (4%), and other (49%). These 4 primary serotypes, which were among the 5 most common among isolates submitted to NARMS overall, accounted for 51% of fully serotyped isolates. Of the 48 states, <2% of isolates were not fully serotyped for 31 states, 2%–8% for 15 states, and >86% for 2 states.

Overall resistance was detected in 2,320 (12%) isolates. Ampicillin-only resistance was the most common pattern, detected in 1,254 (6.5%) isolates, of which 60% were Typhimurium (Table 1; Figure 1). Ceftriaxone/ampicillin resistance was detected in 599 (3.1%) isolates, of which 33% were Newport, 27% Typhimurium, and 15% Heidelberg. Ciprofloxacin nonsusceptibility was detected in 467 (2.4%) isolates, of which 20% were resistant to ampicillin and 45% were Enteritidis. Only 38 (0.2%) isolates were both resistant to ceftriaxone and nonsusceptible to ciprofloxacin; these were included only in the ceftriaxone/ampicillin resistance category. Most isolates with ceftriaxone/ampicillin resistance, ciprofloxacin nonsusceptibility, or ampicillin-only resistance showed resistance to other agents tested (Figure 1) (*9*). The 4 serotypes accounted for 73% of 2,320 isolates with any clinically important resistance. The percentages of isolates with ciprofloxacin nonsusceptibility and ampicillin-only resistance among not fully serotyped isolates were similar to those among all *Salmonella*.

**Table 1:** Nontyphoidal *Salmonella* isolates with clinically important resistance, by serotype and resistance category, United States, 2004–2012*

Resistance category	Typhimurium, no. (%), n = 3,324	Enteritidis, no. (%), n = 3,501	Newport, no. (%), n = 2,175	Heidelberg, no. (%), n = 738	Other fully serotyped, no. (%), n = 9,265	Not fully serotyped, no. (%), n = 407	Total NTS, no. (%), n = 19,410
Cipro†	54 (1.6)	211 (6.0)	7 (0.3)	2 (0.3)	183 (2.0)	10 (2.5)	467 (2.4)
Cef/Amp‡	162 (4.9)	8 (0.2)	198 (9.1)	87 (11.8)	141 (1.5)	3 (0.7)	599 (3.1)
Amp-only§	750 (22.6)	90 (2.6)	25 (1.1)	94 (12.7)	274 (3.0)	21 (5.2)	1,254 (6.5)
Any of the above¶	966 (29.1)	309 (8.8)	230 (10.6)	183 (24.8)	598 (6.5)	34 (8.4)	2,320 (12.0)

*Total NTS isolates include isolates serotyped as Typhimurium, Enteritidis, Newport, and Heidelberg; isolates serotyped as other than these 4; and those not fully serotyped. Amp-only, resistant to ampicillin but susceptible to ceftriaxone and ciprofloxacin; Cef/Amp, resistant to ceftriaxone and ampicillin; Cipro, nonsusceptible to ciprofloxacin but susceptible to ceftriaxone; NTS, nontyphoidal *Salmonella*. Cipro, Cef/Amp, and Amp-only are mutually exclusive categories. †Nonsusceptible to ciprofloxacin (MIC >0.12 μg/mL) but susceptible to ceftriaxone, with or without resistance to other agents. ‡Resistant to ceftriaxone (MIC ≥4 μg /mL) and ampicillin (MIC ≥32 μg /mL), with or without nonsusceptibility to ciprofloxacin or resistance to other agents; of the 599 ceftriaxone-resistant isolates, 38 (0.2% of all NTS isolates) were nonsusceptible to ciprofloxacin. §Resistant to ampicillin but susceptible to ceftriaxone and ciprofloxacin, with or without resistance to other agents. ¶Nonsusceptible to ciprofloxacin, resistant to ceftriaxone, or resistant to ampicillin.

### Surveillance and Resistance Data by State and Year

All 48 states reported *Salmonella* infections to LEDS. Not all states reported infections every year: 47 reported any *Salmonella*, 44 reported Typhimurium, 45 reported Enteritidis, 43 reported Newport, and 39 reported Heidelberg. Many states had wide fluctuations in the annual overall incidence, ranging from 3.1 (Florida) to 28.4 (Mississippi) infections/100,000 person-years.

All 48 states submitted *Salmonella* isolates to NARMS. Not all states submitted isolates every year: 44 submitted any *Salmonella*, 32 submitted Typhimurium, 31 submitted Enteritidis, 23 submitted Newport, and 5 submitted Heidelberg. For Heidelberg and many less common serotypes, small numbers of isolates were tested; in isolates from many states, low or no resistance was detected (e.g., no ceftriaxone resistance among 109 Heidelberg isolates from 19 states). However, very high resistance was assigned to some states for which small numbers were tested (e.g., 1 ceftriaxone-resistant of only 1 tested).

### Model Estimates of Annual Resistance Incidence by State

Rates of *Salmonella* incidence in Florida were much lower than those from its 6 closest states. We adjusted for this finding in the Bayesian hierarchical model (online Technical Appendix). 

**Figure 2:**
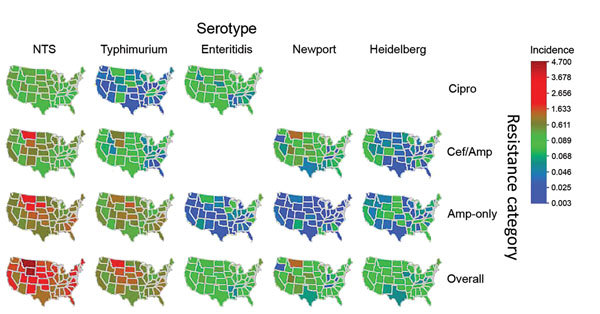
Estimated incidence of infection with all NTS and major serotypes with clinically important resistance (no. infections per 100,000 person-years), by state and resistance category, United States, 2004–2012. Estimates were derived by using Bayesian hierarchical models. All NTS includes the 4 major and other serotypes. Isolates in each category may have resistance to other agents. Data on Cipro among Newport (8 isolates), Cipro among Heidelberg (*7*), and Cef/Amp among Enteritidis (*2*) were too sparse to use in the Bayesian hierarchical models. Overall resistance was defined as Cipro, Cef/Amp, or Amp-only. Amp-only, resistant to ampicillin (MIC >32 μg/mL) but susceptible to ceftriaxone and ciprofloxacin; Cef/Amp, resistant to ceftriaxone (MIC >4 μg/mL) and ampicillin; Cipro, nonsusceptible to ciprofloxacin (MIC >0.12 μg /mL) but susceptible to ceftriaxone; NTS, nontyphoidal *Salmonella*.

For the 48 states, mean resistance incidence, estimated by serotype and resistance category, varied geographically (Figure 2). For all *Salmonella,* rates (infections per 100,000 person-years) ranged as follows: 0.88–4.69 (median 1.81) for overall resistance; 0.45–2.95 (median 0.94) for ampicillin-only resistance; 0.15–2.20 (median 0.38) for ceftriaxone/ampicillin resistance; and 0.11–0.87 (median 0.33) for ciprofloxacin nonsusceptibility. For example, rates of Typhimurium infections with overall resistance were high for many states in the West/Midwest (e.g., Montana, South Dakota, Wyoming, Iowa, Colorado). Rates of Enteritidis infections with ciprofloxacin nonsusceptibility were low for many states in the South (e.g., Mississippi, Arkansas, Louisiana, South Carolina, Alabama).

We observed that the shrinkage of resistance proportions was inversely related to the number of isolates tested, (i.e., more shrinkage with smaller numbers). Examples are shown in the online Technical Appendix.

### Model Estimates of Resistance Incidence Overall

**Figure 3:**
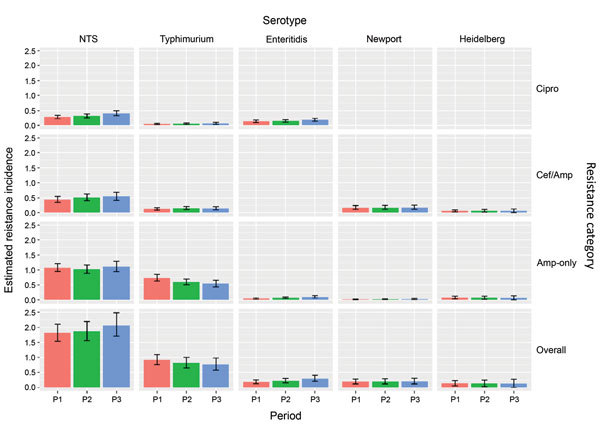
Estimated incidence of NTS infections with clinically important resistance (no. infections/100,000 person-years), by period, serotype, and resistance category, United States, 2004–2012. Estimates were derived by using Bayesian hierarchical models. All NTS includes the 4 major and other serotypes. Three mutually exclusive resistance categories were defined. Isolates in each category may have resistance to other agents. Data on Cipro among Newport (8 isolates), Cipro among Heidelberg (*7*), and Cef/Amp among Enteritidis (*2*) were too sparse to use in the Bayesian hierarchical models. Overall resistance was defined as Cipro, Cef/Amp, or Amp-only. Data were grouped into 3 periods (P): 2004–2006 (P1), 2007–2009 (P2), and 2010–2012 (P3). Error bars indicate 95% credible intervals. Amp-only, resistant to ampicillin (MIC >32 μg/mL) but susceptible to ceftriaxone and ciprofloxacin; Cef/Amp, resistant to ceftriaxone (MIC >4 μg/mL) and ampicillin; Cipro, nonsusceptible to ciprofloxacin (MIC >0.12 μg /mL) but susceptible to ceftriaxone; NTS, nontyphoidal *Salmonella*; P, period.

Resistance incidence rates were relatively stable, and 95% CrIs overlapped substantially for the 3 periods (Figure 3). For overall *Salmonella* infections (Table 2), we estimated the incidence of resistant culture-confirmed infections per 100,000 person-years for 2004–2012 as follows: 1.93 (95% CrI 1.60–2.35) for any clinically important resistance, 1.07 (95% CrI 0.86–1.32) for ampicillin-only resistance, 0.51 (95% CrI 0.35–0.70) for ceftriaxone/ampicillin resistance, and 0.35 (95% CrI 0.24–0.51) for ciprofloxacin nonsusceptibility. Newport, Typhimurium, and Heidelberg accounted for 75% of the incidence of ceftriaxone/ampicillin-resistant infections; Typhimurium accounted for 59% of the incidence of ampicillin-only–resistant infections; and Enteritidis accounted for 45% of the incidence of ciprofloxacin-nonsusceptible infections. Overall, the 4 serotypes accounted for 73% of the incidence of *Salmonella* infections with any clinically important resistance.

## Discussion

This report provides much-needed national incidence estimates for clinically important antimicrobial drug–resistant *Salmonella* infections in the United States. Overall, we estimate the incidence of such culture-confirmed infections to be ≈2/100,000 person-years. Clinically important resistance is strongly linked to specific serotypes. Enteritidis accounts for about half the incidence of ciprofloxacin-nonsusceptible infections; Newport, Typhimurium, and Heidelberg for three fourths of the incidence of infections with resistance to both ceftriaxone and ampicillin; and Typhimurium for more than half the incidence of infections with ampicillin-only resistance. Many of these isolates with clinically important resistance are also resistant to other agents (*8*,*9*). Although these 4 serotypes account for about half of culture-confirmed *Salmonella* infections, they account for nearly three fourths of the incidence of clinically important resistant infections (*6*,*9*). This finding suggests that strategies to reduce the incidence of infections caused by these 4 serotypes could have a larger effect on reducing the incidence of resistant *Salmonella* infections overall.

Using the Bayesian hierarchical model, we improved the estimation of resistance incidence by addressing issues related to missing and sparse state data, particularly for certain combinations of serotypes and resistance. Both surveillance databases showed great variation in reporting by state and year; these variations are probably associated with testing only small numbers of isolates in certain states, underreporting, and incomplete serotyping (*6*,*8*). Therefore, crude estimates based on observed data could lead to biased estimation. We mitigated these issues by statistically borrowing strength from neighboring states and previous years (*22*). We present observed and predicted state resistance incidence estimates by year (online Technical Appendix) to illustrate how our Bayesian hierarchical model smooths state-to-state variability of observed data. We used an estimation scheme called shrinkage, which moved disparate estimates toward a common central value, leading to a more robust set of estimates (*25*). We noted that the shrinkage of resistance proportions was inversely related to the number of isolates tested (online Technical Appendix Figure 1).

Our analysis has limitations. Because LEDS is a passive surveillance system, underreporting probably occurs in most states (*6*); it was marked in Florida, and we adjusted for this only in the Bayesian hierarchical model (online Technical Appendix). We assumed that populations under surveillance are defined by the US Census population data, although populations are mobile and illnesses are sometimes reported by the state in which they are diagnosed rather than the state in which the patient resides (*6*,*21*). The proportion of isolates that were not fully serotyped varied by state and was much higher in LEDS than NARMS. This finding suggests that isolates submitted to NARMS were more likely to be serotyped; regardless, we found similar distributions of major serotypes in LEDS and NARMS. Our approach of imputing missing serotypes of nonserotyped and partially serotyped LEDS isolates by state is reasonable because of the similar distribution of major serotypes in NARMS and LEDS. We did not include serogroup information when imputing partially serotyped isolates; such an approach would not alter our estimates. However, refined methods for imputing partially serotyped isolates could be useful for other analyses.

Because we created mutually exclusive categories, our incidence estimates for ciprofloxacin nonsusceptibility and for ampicillin-only resistance do not include all *Salmonella* with ciprofloxacin nonsusceptibility and ampicillin resistance, respectively. Isolates resistant to ceftriaxone and ampicillin, of which there were many, and those resistant to ceftriaxone and nonsusceptible to ciprofloxacin, were included only in the ceftriaxone/ampicillin resistance category. Furthermore, we do not provide estimates for resistance to trimethoprim-sulfamethoxazole, which can be used for noninvasive infections (*12*); during 2004–2012, <2% of *Salmonella* isolates were resistant to trimethoprim-sulfamethoxazole, 79% of which were also resistant to ceftriaxone or ampicillin, or nonsusceptible to ciprofloxacin (*8*; CDC, unpub. data).

Surveillance data capture culture-confirmed infections only, which represent a fraction of all infections (*6*,*8*,*9*). Our estimates total ≈6,200 culture-confirmed *Salmonella* infections with clinically important resistance annually (*21*). CDC has estimated that for every laboratory-confirmed case of *Salmonella*, there are many other undetected cases; the most recent estimate is 29 infections for every 1 culture-confirmed case (*1*). Because persons with resistant infections are at increased risk for more serious illness that may result in medical attention, such infections may be more likely than susceptible infections to be detected through culture-based surveillance (*15*–*18*,*26*). The ratio of undetected to detected resistant infections has not been estimated.

We found marked state-to-state variation in the incidence of resistant infections. Additional modeling, taking into account the varying distributions of infections by geography, serotype, demographic subgroup, and season, would be needed to help elucidate the reasons (*27*,*28*). Infections among older persons have been associated with increased rates of invasive illness and hospitalization, which may be more likely to be detected; thus, these estimates may represent a higher proportion of older patients than actually exists (*13*,*16*,*21*,*26*). Estimates are based on resistance among all *Salmonella* isolates, which are mostly isolated from fecal samples (*9*). Therefore, these estimates of resistant infections represent mostly noninvasive infections, only a fraction of which may require antimicrobial treatment (*9*,*12*). About 27% of patients with culture-confirmed salmonellosis are hospitalized (*1*). If patients with resistant infections are more likely to be hospitalized, these estimates may disproportionately reflect hospitalized patients (*15*–*18*).

For our estimates, we used data based on current laboratory methods, reporting, and isolate submission practices in states. With increasing use of culture-independent diagnostic tests by clinical laboratories, we anticipate changes in reporting and submission of isolates to public health laboratories (*29*). These changes would warrant model adjustments for future estimation and assessment of changes over time.

Annual NARMS reporting of resistance percentages remains a useful approach for tracking resistance, particularly emerging resistance in serotypes in low numbers of tested isolates (*8*). The method we have developed (using 2 data sources) provides a way to understand changes in the incidence of resistance especially for serotypes like Heidelberg, which is decreasing in incidence but increasing in the proportion resistant to ceftriaxone (*6*,*8*,*9*). By estimating resistance incidence rather than percentage of resistant isolates, we remove a major confounder to interpretation of estimated resistance levels. Our 95% CrIs incorporate uncertainties associated with missing and sparse data. However, our results go a long way toward this understanding. The overlapping 95% CrIs for ceftriaxone-resistant Heidelberg that we found for the 3 periods suggest that incidence rates were relatively stable during 2004–2012. A future, more detailed analysis could assess resistance incidence trends in Heidelberg and other serotypes.

Antimicrobial drug use in food-producing animals is a major driver of—although not the only contributor to—resistant *Salmonella* infections. An example is the contribution of third-generation cephalosporin use in poultry to ceftriaxone resistance among Heidelberg infections of humans (*30*–*32*). FDA has taken actions to contain the spread of antimicrobial-resistant bacteria and prolong the usefulness of antimicrobial agents, including a strategy for limiting antimicrobial use in food animals to therapeutic uses and agents administered under veterinary supervision (*9*,*33*). Even more stringent actions are being applied in the European Union (*9*,*34*). Reservoirs of infection vary by serotype, and resistant infections have been linked to a variety of sources and exposures (*7*,*17*,*35*–*37*). For example, an outbreak of multidrug-resistant (MDR) Typhimurium infections with resistance to ampicillin was linked to consumption of contaminated ground beef (*17*,*35*). MDR Newport infections with resistance to ceftriaxone were linked to exposure to infected dairy cattle and consumption of contaminated ground beef (*14*,*36*). Infections with Enteritidis that are nonsusceptible to ciprofloxacin have been associated with international travel (*37*). Recently, MDR strains of other serotypes, including I 4,[5],12:i:- and Dublin, have become an increasing concern; these serotypes have been linked to swine and cattle sources, respectively (*8*,*38*). NARMS needs to continue to monitor emerging resistance patterns by serotype. The 4 major serotypes that have been driving the incidence of resistant infections should continue to be high priorities in combating resistance.

National incidence estimates of resistant *Salmonella* infections are needed to track progress to support the US President’s Executive Order to combat antibiotic-resistant bacteria (*39*,*40*). Such estimates help define the magnitude of the resistance problem, target prevention efforts, and assess whether control measures are working. Further development of these methods can be used to assess progress from control measures.

**Table 2:** Estimated incidence of nontyphoidal *Salmonella* infections with clinically important resistance, by serotype and resistance category, United States, 2004–2012*

Resistance category	No. infections/100,000 person-years (95% credible interval)*				
	All NTS	Typhimurium	Enteritidis	Newport	Heidelberg
Cipro†	0.35 (0.24–0.51)	0.05 (0.02–0.10)	0.15 (0.09–0.25)	0.005‡	0.002‡
Cef/Amp§	0.51 (0.35–0.70)	0.14 (0.08–0.23)	0.006‡	0.18 (0.08–0.29)	0.06 (0–0.13)
Amp-only¶	1.07 (0.86–1.32)	0.63 (0.43–0.87)	0.08 (0.03–0.16)	0.02 (0.01–0.05)	0.08 (0–0.18)
Any of the above #	1.93 (1.60–2.35)	0.82 (0.61–1.05)	0.24 (0.14–0.38)	0.20 (0.11–0.32)	0.14 (0.002–0.28)

*Estimates and 95% credible intervals were derived by using Bayesian hierarchical models. Cipro, Cef/Amp, and Amp-only are mutually exclusive categories. Estimates for any clinically important resistance were derived by summing estimates for the mutually exclusive categories. Serotypes other than Typhimurium, Enteritidis, Newport, and Heidelberg were combined in an “other” category. For all NTS, estimates were derived by summing those derived for the 4 major serotypes and other category. Amp-only, resistant to ampicillin but susceptible to ceftriaxone and ciprofloxacin; Cef/Amp, resistant to ceftriaxone and ampicillin; Cipro, nonsusceptible to ciprofloxacin but susceptible to ceftriaxone; NTS, nontyphoidal *Salmonella*. †Nonsusceptible to ciprofloxacin (MIC >0.12 μg/mL) but susceptible to ceftriaxone, with or without resistance to other agents. ‡Only 8, 7, and 2 isolates of Enteritidis, Newport, and Heidelberg, respectively, showed this resistance pattern; thus, state-year data were too sparse to use in the Bayesian hierarchical models. Crude estimates are shown, calculated as mean incidence for the serotype multiplied by mean resistance proportion over the 9 y. §Resistant to ceftriaxone (MIC >4 μg /mL) and ampicillin (MIC >32 μg /mL), with or without nonsusceptibility to ciprofloxacin or resistance to other agents. ¶Resistant to ampicillin but susceptible to ceftriaxone and ciprofloxacin, with or without resistance to other agents. #Nonsusceptible to ciprofloxacin, resistant to ceftriaxone, or resistant to ampicillin.

Technical Appendix Bayesian hierarchical model for modeling spatiotemporal patterns of antimicrobial drug resistance rates.
