# Supplementary material for: Estimated Incidence of Antimicrobial Drug–Resistant Nontyphoidal Salmonella Infections, United States, 2004–2012
Source: Emerg Infect Dis. 2017 Jan;23(1):29–37. doi: 10.3201/eid2301.160771 (PMC5176233; doi:10.3201/eid2301.160771)
Supplement: Technical Appendix — Bayesian hierarchical model for modeling spatiotemporal patterns of antimicrobial drug resistance rates. [file 16-0771-Techapp-s1.pdf]

Article DOI: http://dx.doi.org/10.3201/eid2301.160771

Estimated Incidence of Antimicrobial Drug–Resistant Nontyphoidal *Salmonella* Infections, United States, 2004–2012

Technical Appendix

Background

We describe the use of a Bayesian hierarchical model (BHM) to estimate resistance incidence. We used data on isolations of *Salmonella* serotypes from the Laboratory-based Enteric Disease Surveillance (LEDS) and resistance proportions from the National Antimicrobial Resistance Monitoring System (NARMS). The yearly surveillance data of 48 states (excluding Alaska and Hawaii) from both LEDS and NARMS are volatile due to sampling variation and may be biased due to underreporting. For NARMS data, many states have small numbers of isolates due to the sampling scheme (1 in 20), particularly for Heidelberg and less common serotypes. The estimation of resistance proportions by state and year is unreliable due to the small sample size. BHM provides a framework to mitigate the issues based on partial pooling (borrowing strength) from structured data, e.g. neighboring states may exhibit similarity in incidence and resistance proportions. BHM reduces variability in estimates by spatial smoothing of geographically related surveillance data. It provides a flexible approach by accounting for structured and non-structured variances in the data.

Another advantage of BHM is its utility in handling missing data. Data were missing from both surveillance systems, especially for some combinations of serotypes and resistance types. For example, not all states reported or submitted isolates of the major serotypes every year, thus infection incidence rates and resistance proportions were not available for the states that did not report or submit isolates for the year. In Bayesian statistics, missing values are treated as unknown parameters and are estimated in the same manner as other parameters in the model, and Bayesian estimation of missing values takes into account the uncertainty of parameter estimation.

Bayesian hierarchical model

NARMS model of resistance proportion:

We assume that the observed number of resistant isolates follows a binomial distribution with unknown proportion parameter $\theta_{s,t}$

$$n_{s,t} \sim bin(\theta_{s,t}, T_{s,t})$$

where $n_{s,t}$ is the number of isolates resistant to the antimicrobial drug in state *s* and time *t*, $T_{s,t}$ is the number of isolates tested in state *s* in time *t*. and $\theta_{s,t}$, the unknown probability of the resistance in state *s* and period *t*.

We use the logit link function to relate the probability of resistance in a state and year to predictive factors

$$\log[(\theta_{s,t})/(1-\theta_{s,t})] = \alpha + v_{s,t} + u_{s,t} + \varphi_{s,t} \quad (1)$$

where α is a random effect of grand mean,

$$\alpha \sim N(0, \tau_{\alpha})$$

$v_{s,t}$ represents temporal autocorrelation of random walk, i.e. the value at time *t* were related to the previous value at time *t*-1 with random drift specified by variance parameter [illegible]

$$v_{s,1} \sim N(0, \tau_v)$$

$$v_{s,t} \sim N(v_{s,t-1}, \tau_v)$$

We set the normal distribution variance parameter, [illegible] equal to 2 to impose a temporal autocorrelation between the resistance proportion of a state in a given year and that of the preceding year; that of the first year is set to be normal variate of zero mean to anchor the posterior.

$u_{s,t}$ in equation 2 is the structured state spatial random effect reflecting a time-varying neighborhood effect (*2*).

$$u_{s,t} | u_{-s,t} \sim N(\overline{u_{s,t}}, \frac{1}{\tau_u m_s})$$

where u $_{-s}$ denotes states adjacent to state *s*. Adjacency is defined as sharing a border with the focal state *s*, $\overline{u_{s,t}}$ is the mean of estimates across the neighbors of state *s* at time *t*, and m_s is

the number of neighboring states of state s. For τ_u ,we adopted a weak gamma prior proposed by Kelsall and Wakefield (*1*)

$$\tau_u \sim G(0.5, 0.0005)$$

This prior assumes that the spatial random effects for a single adjacent state has a standard deviation centered around 0.05 with 1% probability being smaller than 0.01 or larger than 2.5 (*1*).

Finally, $\varphi_{s,t}$ is state-time interaction term of normal variate

$$\varphi_{s,t} \sim N(0, \tau_\varphi)$$

After experimenting with different options, we settled with a fixed τ_φ equal to 2 to balance the amount of shrinkage from observed values across the various states and years. For missing $T_{s,t}$, we assumed them as either the mean of the known submission rates (estimated from submitted rates over the years when submission occurred) or as 1 if the former was not available. In the latter case, the influence of the assumed values (one isolate) would be minimized.

LEDS model of *Salmonella* incidence:

The standard model for incidence based on count data is the Poisson distribution (3). However, counts and incidence rates of different serotypes varied drastically from year to year (Fig. 2). We found that use of a Poisson model was inadequate to capture the variability observed in the data and resulted in estimates of little, if any, shrinkage of observed values. To capture the observed variability in yearly observed incidence rates, we adopted a truncated normal distribution for the incidence rates (/100,000) $I_{s,t}$ (truncated for $I_{s,t} < 0$)

$$I_{s,t} \sim N(\mu_{s,t}, 0.1)$$

We adopted a similarly structured model as the NARMS model described above

$$\mu_{s,t} = \alpha + v_{s,t} + u_{s,t} + \varphi_{s,t}$$

We used following priors for the parameters

$$\alpha \sim N(0, 0.5)$$

$v_{s,\,t}$ was temporal autocorrelation of random walk

$$v_{s,1} \sim N(0, \tau_v)$$

$$v_{s,t} \sim N(v_{s,t-1}, \tau_v)$$

We set τ_v as 5 to impose a temporal autocorrelation of incidence rates of state s to be related to that of the preceding year; that of the first year was set to be normal variate of zero mean.

$$u_{s,t} | u_{-s,t} \sim N(\overline{u_{s,t}}, \frac{1}{\tau_u m_s})$$

$$\tau_u \sim G(0.5, 0.0005)$$

$$\varphi_{s,t} \sim N(0, 5)$$

Adjustment for not fully serotyped LEDS data

We applied serotype-resistance data to all LEDS isolates, including not fully serotyped isolates, after adjustment for incomplete serotyping for all 48 states. For each state, we imputed serotypes for LEDS isolates that were not fully serotyped based on the observed proportions of five serotype categories (Enteritidis, Typhimurium, Newport, Heidelberg, and other) among fully serotyped isolates over the 9 years.

Adjustment for underreporting to LEDS by Florida

The reported *Salmonella* incidence rates in Florida were much lower than those from states in the region, indicating significant underreporting from the state. We only adjusted for underreporting by Florida for overall nontyphoidal *Salmonella* and the four major serotypes. Table 1 presents means of incidence rates in Florida compared with those in six closest southern states (Alabama, Georgia, Mississippi, South Carolina, North Carolina, and Tennessee) for nontyphoidal *Salmonella* and four major serotypes. To reduce bias in plausible underreporting of incidence data by Florida, we adopted a regional BHM to estimate Florida incidence rates with adjustment for underreporting. The BHM for the region including Alabama, Florida, Georgia, Mississippi, South Carolina, North Carolina, and Tennessee was:

$$\mu_{s,t} = \alpha + v_s + u_t + \varphi_{s,t}$$

where v_s denotes the state effect, u_t the year effect, and $\varphi_{s,t}$ the state-year interaction. The following priors were used

$$\alpha \sim N(0, 0.01)$$

$v_s \sim N(0, 0.1)$

$u_t \sim N(0, 0.1)$

$\varphi_{s,t} \sim N(0, 10)$

Note, we used a large value 10 as the precision parameter for $\varphi_{s,t}$ to shrink Florida estimates more effectively toward the regional mean.

The adjusted estimates of incidence rates in Florida were closer to the means from the six southern states. We used the adjusted incidence rates in Florida (Appendix Table) to replace the observed values as inputs to run the BHM for estimating resistance incidence.

Summary posterior estimates of overall nontyphoidal *Salmonella*:

Posterior estimates of resistance proportion, incidence rates, and resistance incidence of overall nontyphoidal *Salmonella* were derived from the aggregated joint distributions of posterior estimates of the corresponding measures of its component serotypes (Enteritidis, Typhimurium, Newport, Heidelberg, and other). The posteriors of resistance proportions were derived by averaging the predicted numbers of resistant isolates of the serotypes weighted by the numbers of submitted isolates, while the posteriors of incidence rates and resistance incidence rates were derived by summing the posterior estimates of the corresponding measures of the component serotypes.

Summary posterior estimates of clinically important resistance:

Similarly, the posteriors of clinically important resistance for four serotype categories (Enteritidis, Typhimurium, Newport, Heidelberg) were derived from the aggregated joint distributions of the posteriors of the corresponding measures of the mutually exclusive resistance categories (i.e., resistance to ceftriaxone, nonsusceptibility to ciprofloxacin, and resistance to ampicillin).

Posterior estimates vs. observed values:

We assessed the shrinkage of posterior resistance proportions (predicted) vs. crude proportions (observed) related to the number of isolates tested. Appendix Figure 1 shows the shrinkage for ampicillin resistance among isolates of overall nontyphoidal *Salmonella*, isolates of the four major serotypes, and other fully serotyped isolates. As part of model fitting, we plotted predicted estimates and observed values of resistance proportion, *Salmonella* infection incidence,

and resistance incidence by state-year for each of the four major serotypes by resistance category. Predicted estimates vs. observed values for ampicillin resistance among *Salmonella* ser. Typhimurium are shown in Appendix Figures 2–4.

Software

The models were run in R (*4*) with R2WinBUGS package (*5*) calling WinBUGS (*6*), which used Gibbs sampler for estimation of posteriors using Markov chain Monte Carlo MCMC simulation. Three chains of independent starting values of precision parameters were used. After throwing away 5000 burn-ins, 5000 posterior samples of parameters were harvested.

References

1. Kelsall J, Wakefield J. Discussion of Bayesian models for spatially correlated disease and exposure data. In: Sixth Valencia International Meeting on Bayesian Statistics, 1999.

2. Thomas A, Best N, Lunn D, Arnold R, Spiegelhalter D. Appendix 1: Technical details of structured multivariate Gaussian and conditional autoregressive (CAR) models and hyperprior specification. In: GeoBUGS User Manual version 12. 2004.

3. Best N, Richardson S, Thomson A. A comparison of Bayesian spatial models for disease mapping. Stat Methods Med Res. 2005;14:35–59. PubMed http://dx.doi.org/10.1191/0962280205sm388oa

4. R Development Core Team. R: A language and environment for statistical computing. R Foundation for Statistical Computing. 2011.

5. Sturtz S, Ligges U, Gelman A. R2WinBUGS: A package for running WinBUGS from R. J Stat Softw. 2005;12:1–16. http://dx.doi.org/10.18637/jss.v012.i03

6. Lunn DJ, Thomas A, Best N, Spiegelhalter D. WinBUGS—A Bayesian modelling framework: concepts, structure, and extensibility. Stat Comput. 2000;10:325–37. http://dx.doi.org/10.1023/A:1008929526011

Technical Appendix Table. Mean annual incidence rates (per 100,000 person-years) of infections caused by nontyphoidal *Salmonella* (NTS) overall and four major serotypes in Florida and six closest southern states, 2004–2012

State	All NTS	Typhimurium	Enteritidis	Newport	Heidelberg
Other southern states (crude)*	22.08	3.62	2.27	3.73	0.46
Alabama (crude)	20.15	4.13	1.93	2.53	0.54
Georgia (crude)	24.96	3.06	2.30	4.21	0.58
Mississippi (crude)	28.38	5.51	1.43	5.10	0.38
North Carolina (crude)	21.63	3.67	3.07	4.30	0.40
South Carolina (crude)	24.00	2.87	3.31	4.42	0.38
Tennessee (crude)	13.34	2.50	1.58	1.80	0.46
Florida (crude)	3.14	0.13	0.12	0.12	0.02
Florida (adjusted)†	11.90	1.93	1.26	1.87	0.40

*Mean annual incidence rates for six closest southern states, Alabama, Georgia, Mississippi, North Carolina, South Carolina, and Tennessee
†Annual incidence rates adjusted for incomplete serotyping and underreporting replaced observed values in the Bayesian hierarchical model for estimating resistance incidence.

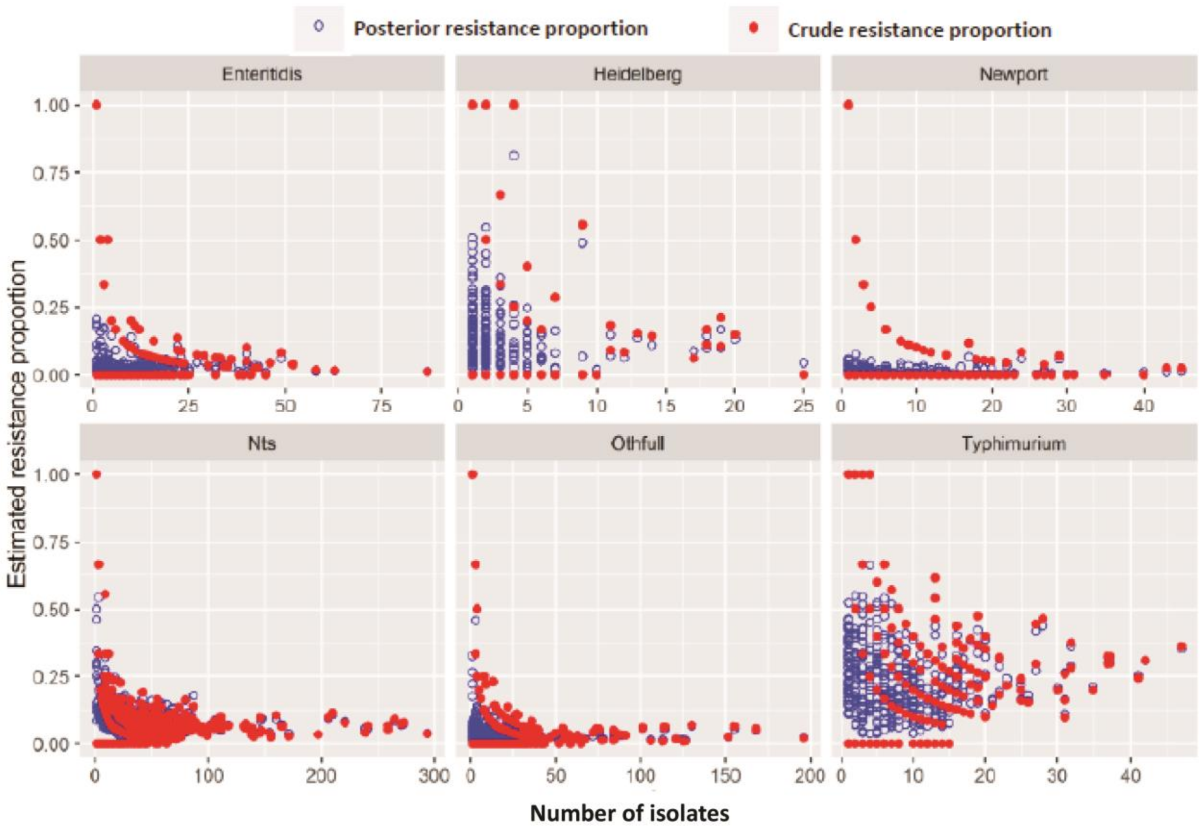

Technical Appendix Figure 1. Shrinkage of posterior estimates and crude proportions of ampicillin resistance among isolates of the 4 major serotypes, isolates of all nontyphoidal *Salmonella* (NTS) serotypes, and other fully serotyped isolates (Othfull), related to the number of isolates tested, by state and year, 2004–2012

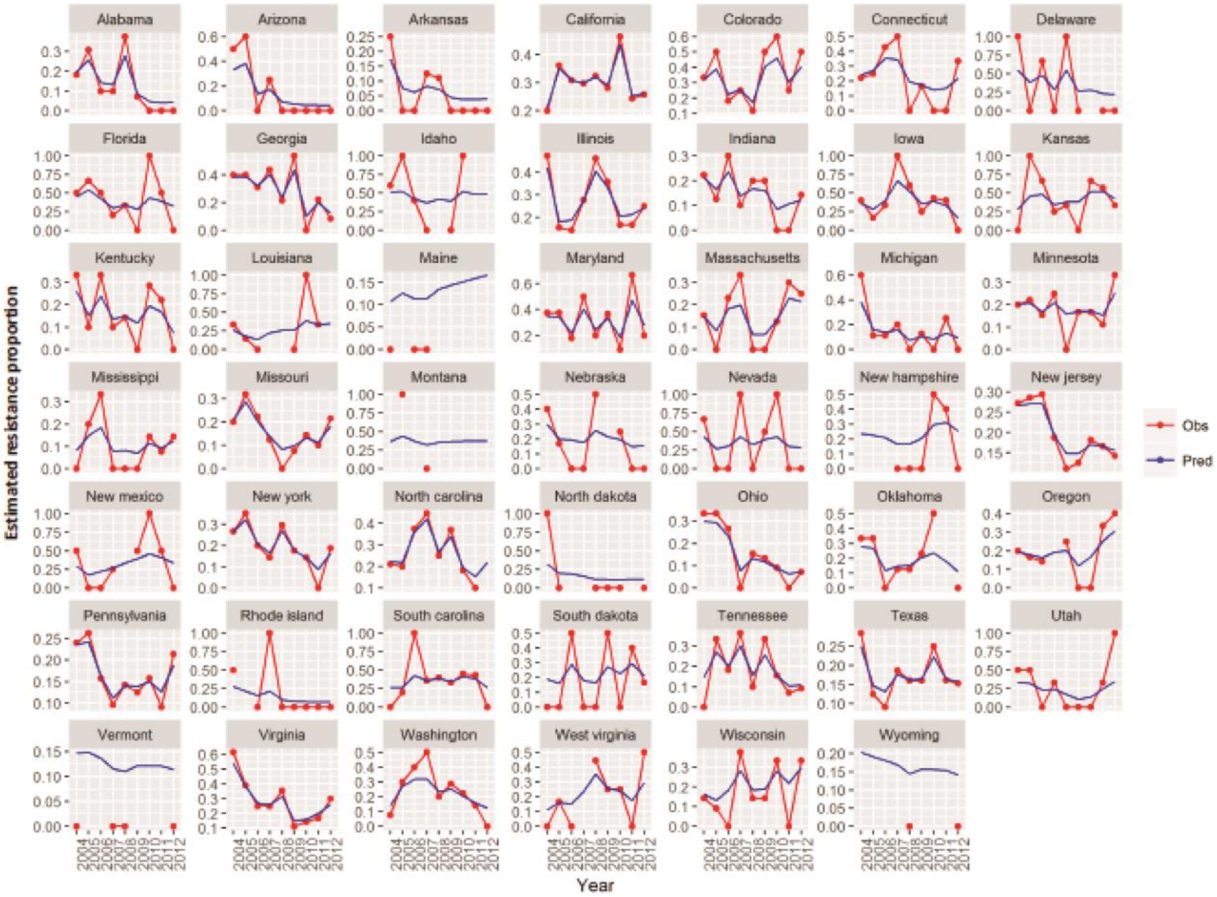

Technical Appendix Figure 2. Comparison of posterior estimates (Pred) and crude proportions (Obs) of ampicillin resistance among *Salmonella* ser. Typhimurium isolates, by state and year, 2004–2012

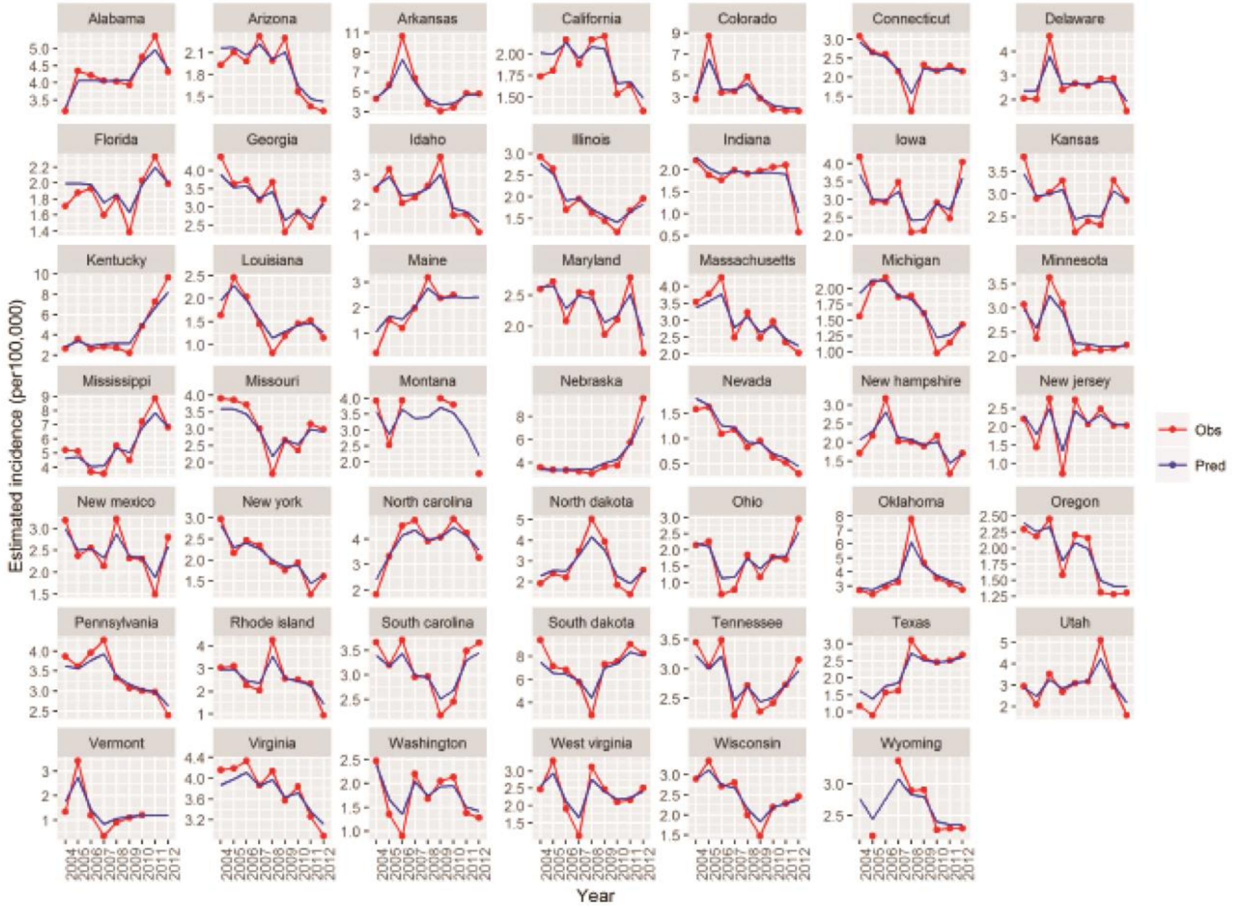

Technical Appendix Figure 3. Comparison of posterior incidence estimates (Pred) and crude incidence rates (Obs) of *Salmonella* ser. Typhimurium infections (per 100,000 person-years), by state and year, 2004–2012

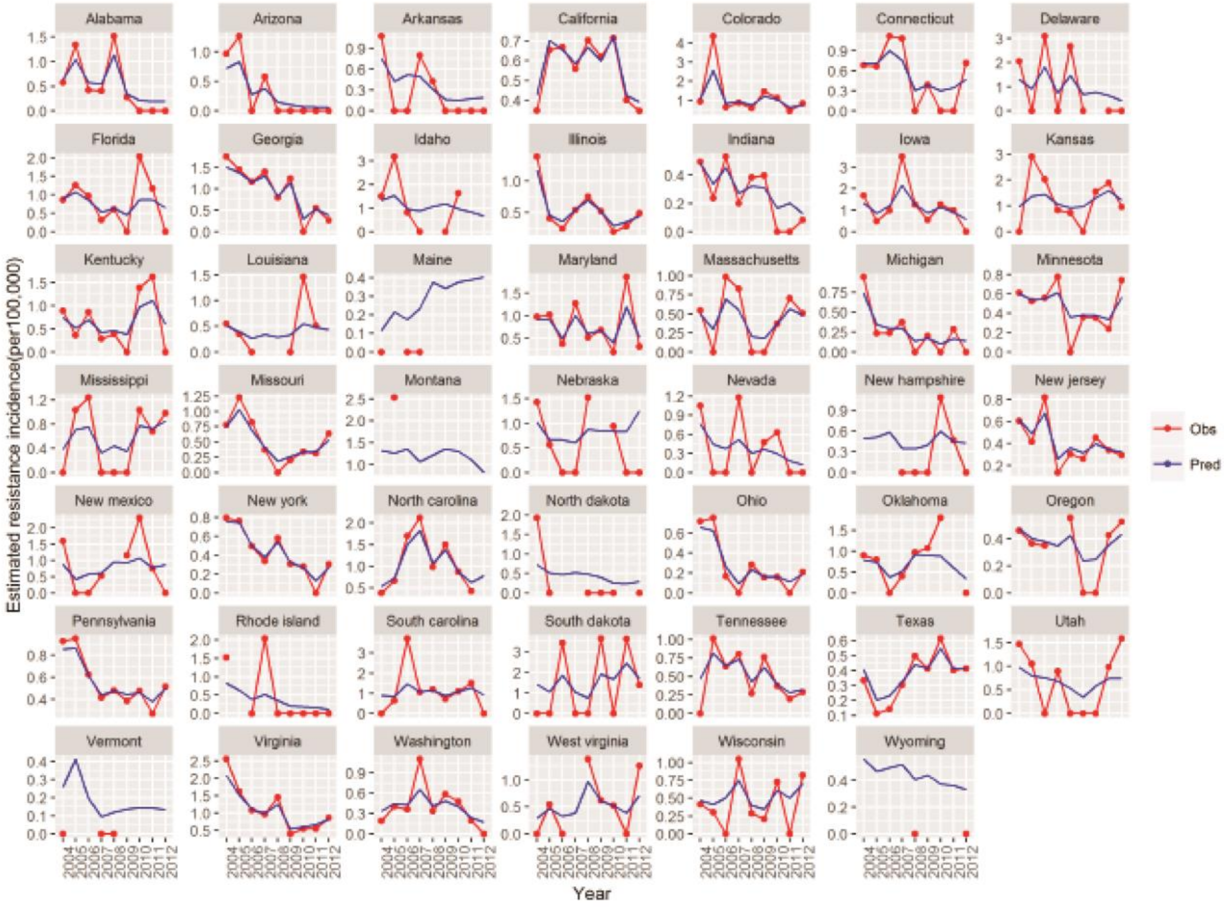

Technical Appendix Figure 4. Comparison of posterior incidence estimates (Pred) and crude incidence rates (Obs) of ampicillin-resistant *Salmonella* ser. Typhimurium infections (per 100,000 person-years), by state and year, 2004–2012
